# Combination Therapy of Albendazole and Praziquantel for Treatment of Cardiac and Multiorgan Hydatid Cyst: A Case Report of Novel Treatment and Literature Review

**DOI:** 10.1002/ccr3.9610

**Published:** 2024-11-29

**Authors:** Soroush Najdaghi, Azin Alizadehasl, Narguess Abbaszade, Seyedeh Fatemeh Hosseini Jebelli, Delaram Narimani Davani, Azam Yalameh Aliabadi, Mehrdad Haghazali, Alireza Yaghoubi Gloverdi

**Affiliations:** ^1^ Heart Failure Research Center, Cardiovascular Research Institute Isfahan University of Medical Science Isfahan Iran; ^2^ Cardio‐Oncology Research Center Rajaie Cardiovascular Medical and Research Institute Tehran Iran; ^3^ Rajaie Cardiovascular Medical and Research Center Iran University of Medical Sciences Tehran Iran

**Keywords:** albendazole, cardiac hydatid cysts, case report, echinococcosis, echocardiography, praziquantel

## Abstract

Cardiac hydatid cysts (CHC) are rare complications of echinococcosis, often presenting diagnostic and therapeutic challenges. We report a case of recurrent CHC in a 35‐year‐old male with a history of cerebral and pelvic hydatid cysts. Diagnostic imaging revealed significant cardiac involvement, necessitating surgical intervention. Postoperative management included a combination of albendazole and praziquantel, resulting in marked improvement during follow‐up. This case highlights the complexities of treating recurrent CHC and suggests that combination therapy may offer benefits in managing extensive cysts. Further research is needed to refine treatment strategies for such cases.

## Graphical abstract

A schematic of a patient's follow‐up with recurrent multi‐organ hydatid cysts, who, due to lack of response to brain and heart surgeries, underwent a trial treatment with praziquantel and albendazole. Six‐month follow‐up shows a reduction in the size and number of cardiac and abdominal cysts.

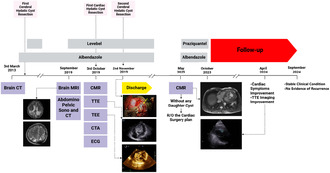

**Abbreviations**
AF: Atrial Fibrillation
CAD: Coronary Arterial Disease
CHCs: Cardiac Hydatid Cysts
CMR: Cardiac Magnetic Resonance Imaging
CT: Computed Tomography
ECG: Electrocardiogram
EF: Ejection Fraction
ELISA: Enzyme‐Linked Immunosorbent Assay
IABP: Intra‐aortic Balloon Pump
IgG: Immunoglobulin G
LV: Left Ventricle
LVH: Left Ventricular Hypertrophy
MR: Mitral Regurgitation
MRI: Magnetic Resonance Imaging
PAIR: Puncture, Aspiration, Injection, and Reaspiration
RV: Right Ventricle
SPAP: Systolic Pulmonary Artery Pressure
TEE: Transesophageal Echocardiography
TTE: Transthoracic Echocardiography
UFH: Unfractionated Heparin
VSDs: Ventricular Septal Defects

**Summary**

- This unique case of recurrent multiorgan hydatid cysts, including cardiac involvement, showcases the successful use of albendazole and praziquantel as a non‐surgical alternative.
- The patient's primary concerns were dyspnea and atypical chest pain, with imaging revealing significant cardiac and pelvic cysts.
- Combination therapy led to cyst reduction, avoiding high‐risk surgery.

## 1 Introduction

Cardiac hydatid cysts (CHCs) are a rare manifestation, comprising approximately 0.5%–2% of all hydatid disease cases [1]. This rarity is due to the inhibitory effect of cardiac contractility on viable cyst presence [2]. This infection is caused by the larval stage of Echinococcus tapeworms, notably Echinococcus granulosus and Echinococcus multilocularis [1].

Demographic patterns of CHCs vary in published studies, with some indicating male incidence and others suggesting a slight female predominance [3, 4]. However, research predominantly points to a higher incidence among young adults, particularly in their third and fourth decades, necessitating further investigation [5].

Clinical presentations of CHCs are variable and often nonspecific, posing diagnostic challenges, especially with concurrent cysts in cardiac, cerebral, and intra‐abdominal regions. While liver and lung cysts are more prevalent (in 65% and 12% of cases, respectively), cardiac involvement typically presents with atypical chest pain, dyspnea, and normal sinus rhythm, in clinical and electrocardiogram (ECG) findings [6]. However, in a few studies, features such as atrial fibrillation (AF) arrhythmia and inverted T waves at the time of admission have been reported [5, 7].

Diagnosis and localization of CHCs rely primarily on noninvasive methods such as serological tests, notably Enzyme‐Linked Immunosorbent Assay (ELISA), for specific antibodies against Echinococcus granulosus [5]. Echocardiography aids in early visualization and characterization of cystic structures within the heart. Computed Tomography (CT) and Cardiac Magnetic Resonance Imaging (CMR) imaging provide detailed anatomical delineation for surgical planning [8].

Although surgical removal is the main treatment strategy, its feasibility depends on cyst accessibility and patient‐specific considerations such as comorbidities. In high‐risk cases or multiple organ involvement, medical therapy may be explored [9]. Medications are commonly used from the benzimidazoles family including albendazole and mebendazole; however, praziquantel showing promise in combination therapy in animal and human studies, although further research is needed [9].

Simultaneous Cerebral hydatid cyst involvement is uncommon, with cysts most frequently located in the parietal lobe. Presentations are mainly non‐specific including symptoms and signs of intracranial hypertension. Moreover, studies report a 36% frequency of patients presenting with seizures, raising the need for preventative anti‐seizure management. The primary treatment is surgical excision, aiming for complete removal without rupture, typically achieved using Dowling's technique. Anti‐helminthic therapy, such as benzimidazoles, is used as an adjunct in cases of systemic involvement, recurrence, or cyst rupture [10].

To the best of our knowledge, our case is the first study that presents recurrent CHCs in a patient with previous cerebral and intrapelvic hydatid cysts, investigating the efficacy of combinational treatment with albendazole and praziquantel over 11 months of follow‐up.

## 2 Case Presentation

On 15th May 2023, a 35‐year‐old male was referred to our hospital, presenting with dyspnea and atypical chest pain, devoid of any lateralized cerebral symptoms, for 1 week before admission. He had no history of smoking, drug use, or congenital anomalies in the family. Upon referral to our hospital, further evaluation was warranted.

### 2.1 Medical Background

**FIGURE 1:**
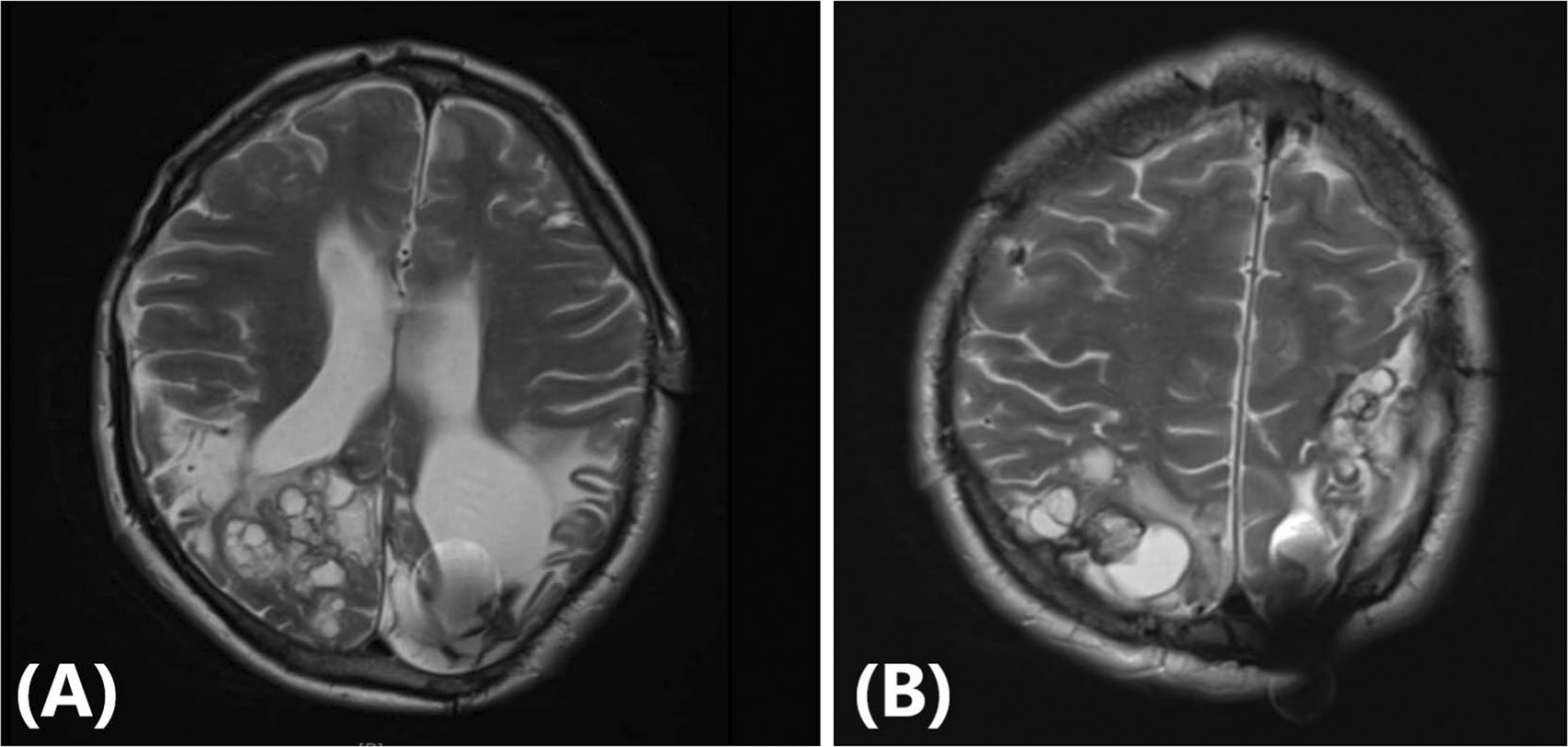
Axial T2‐weighted Brain MRI of a 35‐year‐old Male with Multi‐Organ Hydatid Cyst. (A) There are a few clusters of varying size cysts with high internal signal and thin hypo‐signal wall and without solid components associated with surrounding vasogenic edema at white matter of the right occipital lobe with pressure on the occipital horn of the right lateral ventricle (cut level: occipital horns of the lateral ventricles). (B) Multiple hydatid cysts are visible in the right occipital lobe, characterized by hyperintense signals with thin hypointense walls. The largest cyst exerts a mass effect on the occipital horn of the right lateral ventricle. Significant surrounding vasogenic edema is also noted (cut level: posterior periventricular white matter and occipital lobe). MRI, magnetic resonance imaging.

A review of the patient's medical history revealed no coronary artery disease (CAD) risk factors or significant family history. However, a decade prior, on 3rd March 2013, he was diagnosed with a cerebral hydatid cyst upon brain CT scan (not available for sharing), which manifested through headaches and unilateral paralysis. Surgical cyst resection followed by albendazole therapy at 400 mg twice daily for 6 months had been initiated. Six years later, on September 12, 2019, recurrent cerebral hydatid cysts were identified (Figure 1A,B), prompting consultation with a neurosurgeon, who recommended craniotomy and hydatid cyst removal.

### 2.2 Previous Diagnostic Findings

**FIGURE 2:**
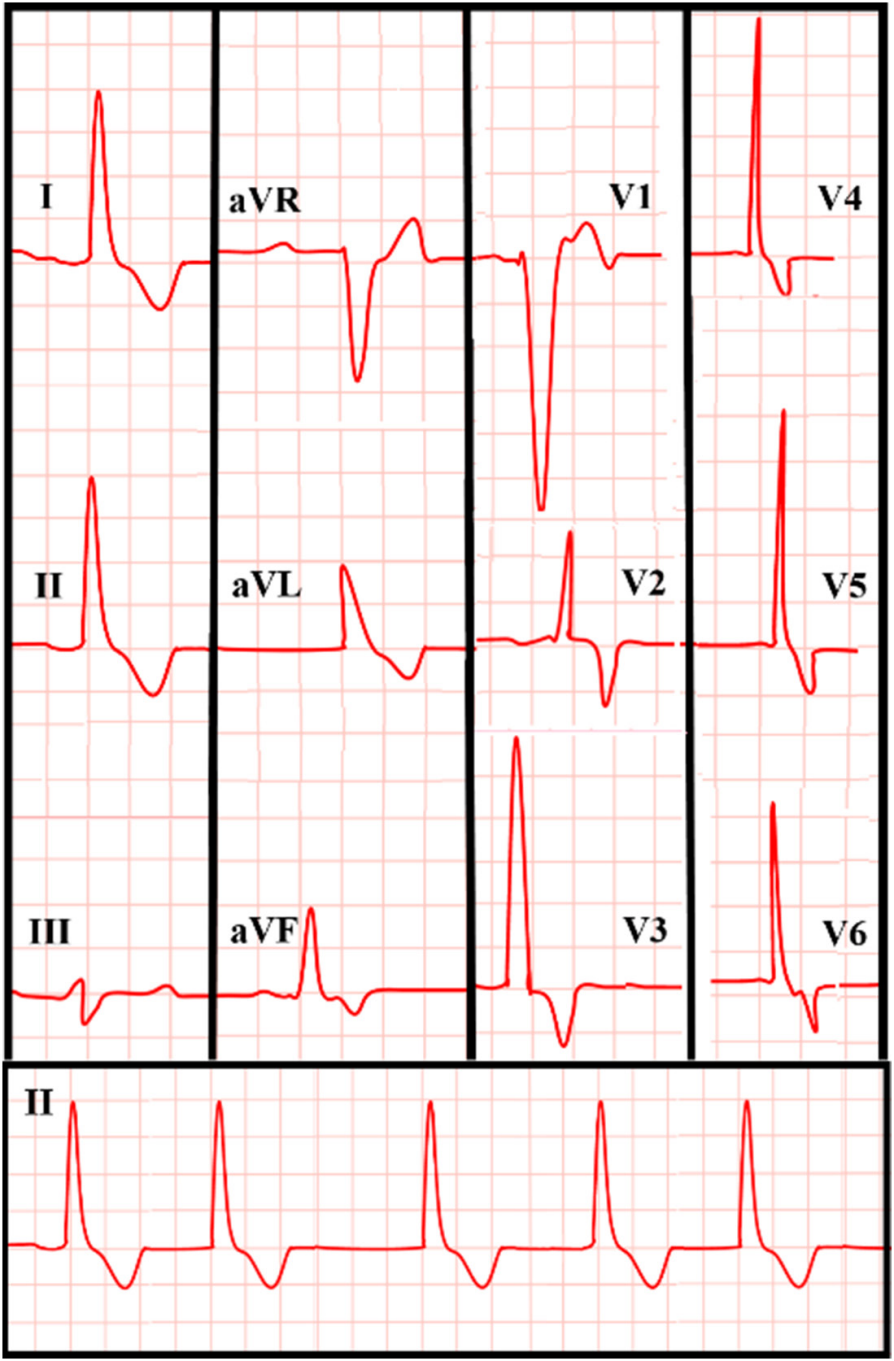
ECG of a 35‐year‐old male with cardiac and multi‐organ hydatid cyst. This ECG reflects AF rhythm with irregular, rapid, and uncoordinated atrial activity visible across the leads. Evidence of LVH is suggested by the large QRS complexes, particularly in the precordial leads. Negative T waves are apparent in most leads, except in leads V1 and aVR, indicating repolarization abnormalities consistent with LVH. AF, atrial fibrillation; ECG, electrocardiogram; LVH, left ventricular hypertrophy.

**FIGURE 3:**
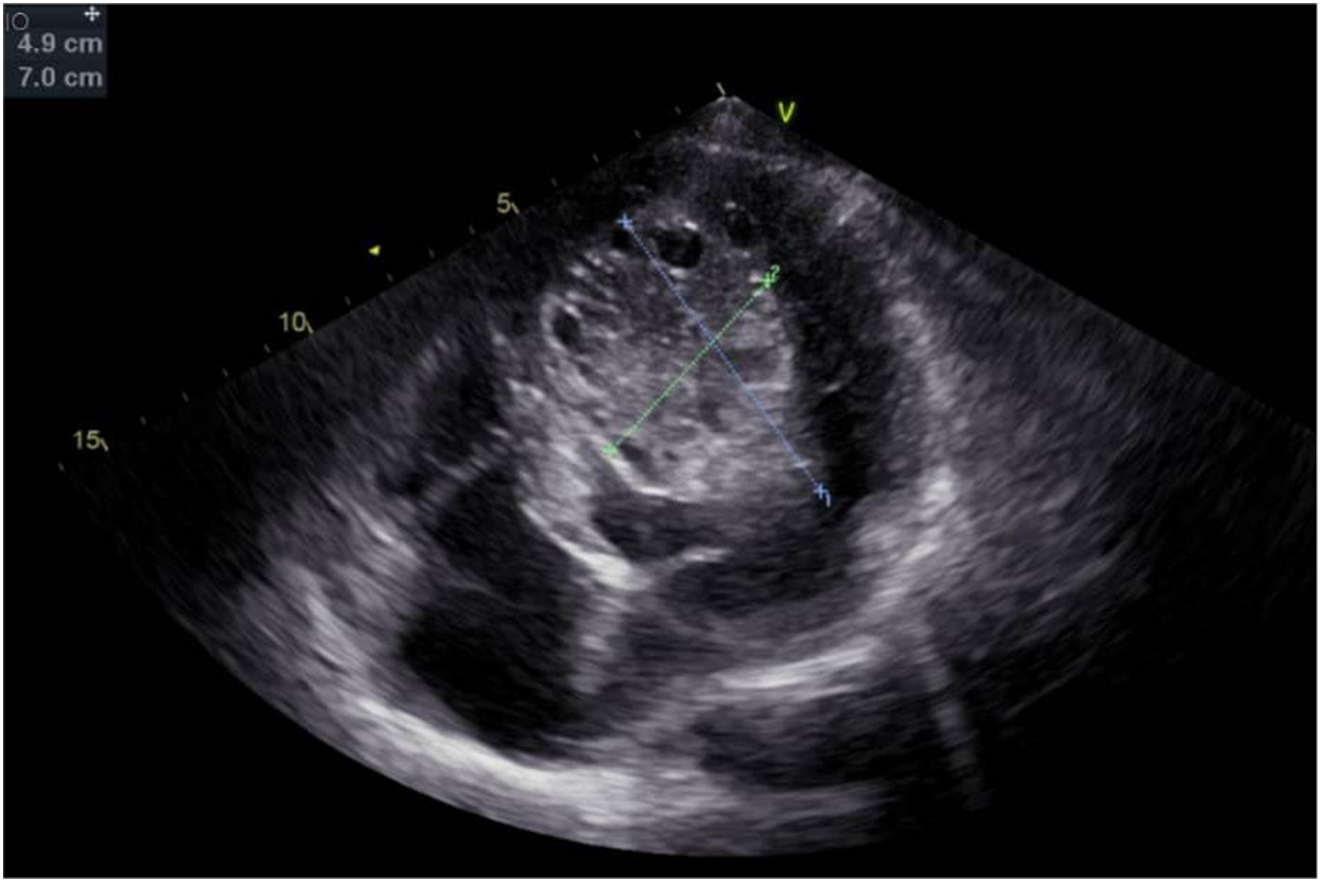
Four chamber TTE of a 35‐year‐old male with multi‐organ hydatid cyst. Severe LV enlargement with a septated echo‐free space (6.1 × 4.3 cm) in the LV cavity with mobile particles on its surface. LV, left ventricle; TTE, transthoracic echocardiography.

**FIGURE 4:**
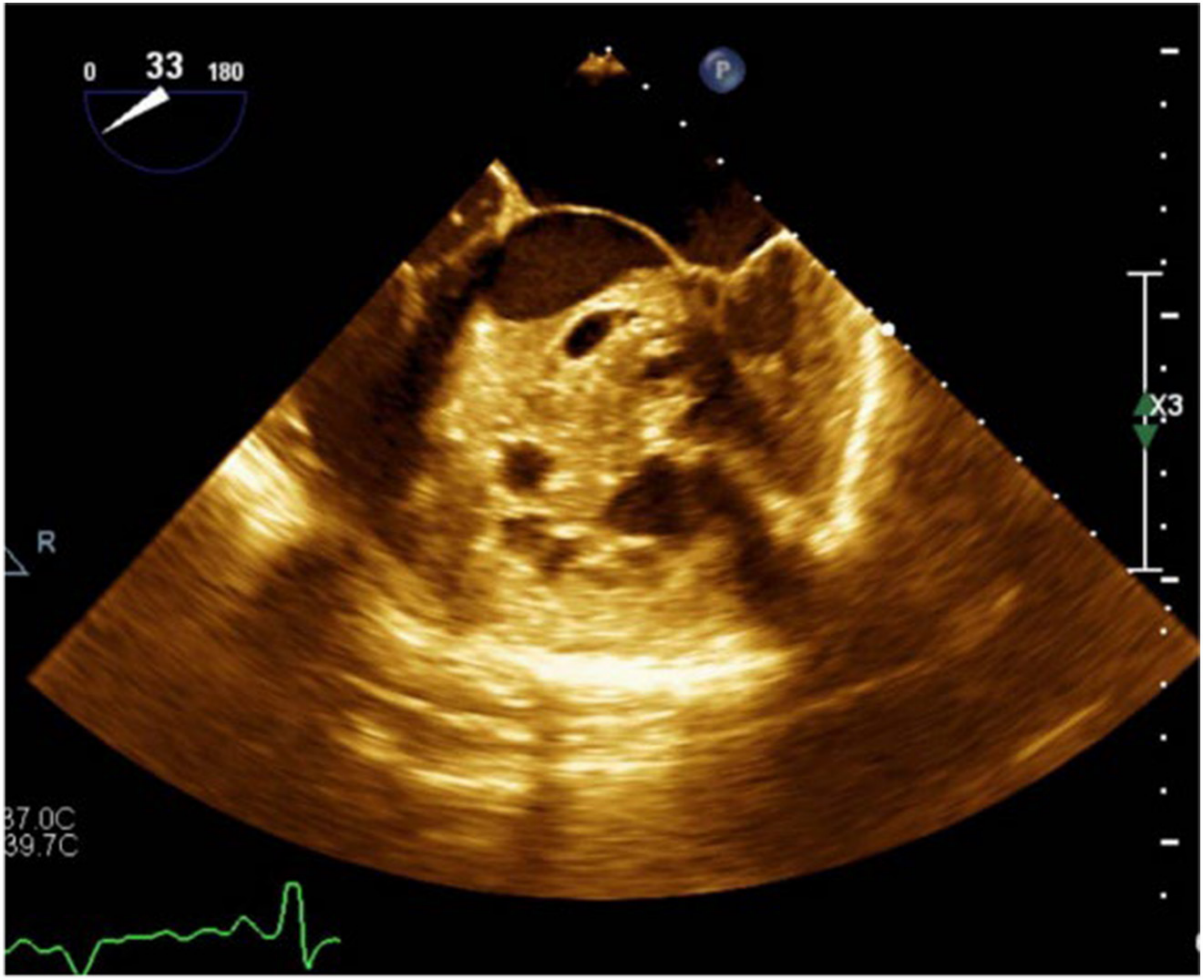
TEE of LV cavity of a 35‐year‐old male with multi‐organ hydatid cyst. TEE shows a Large multi‐lobulated and multi‐cystic mass with septations in some of the cysts and a water‐lily pattern in at least two cysts attached to the septum. LV, left ventricle; TEE, transesophageal echocardiography.

Presurgical comprehensive cardiac assessments (September 2019) revealed AF rhythm, left ventricular hypertrophy (LVH), and negative T waves evident on ECG leads, except in leads V1 and aVR (Figure 2). Transthoracic echocardiography (TTE) unveiled severe left ventricular enlargement with a moderate‐sized echo‐free space within the left ventricular (LV) cavity, bearing a mobile particle suggestive of a residual LV hydatid cyst (Figure 3). Transesophageal echocardiography (TEE) confirmed the TTE findings, indicating severe LV enlargement with moderate systolic dysfunction and a large multi‐cystic mass attached to the septum, occupying a significant portion of the LV cavity (Figure 4). Due to the urgent need for cardiac and brain surgery, preoperative stabilization was considered by administrating metoprolol IV, starting with 5 mg every 5 min for up to three doses, followed by oral administration of 25–50 mg every 12 h. Given the presence of AF and the risk of thromboembolism, anticoagulation is crucial, that had been administrated with an initial bolus of unfractionated heparin (UFH; 60–80 units/kg IV), and discontinued 4–6 h before surgery to minimize bleeding risk.

### 2.3 Primary Cardiac Surgery and the Second Brain Surgery

After consultation with a cardiologist, cardiac surgeon, and neurosurgeon, the consensus was to prioritize cardiac stabilization before brain surgery, given the risks associated with cerebral craniotomy in an unstable cardiac condition. Consequently, on October 3, 2019, the patient underwent intracardiac cyst removal with cardiopulmonary bypass (CPB). During the surgery, multiple cysts were found in the right ventricle (RV), including daughter cysts, collectively equivalent to the size of a small heart. After removing the cysts, a hypertonic saline solution was instilled into the residual cavity to prevent local parasite dissemination. Unexpectedly, a cyst was also discovered within the LV, necessitating a repeat cardiac arrest for careful extraction. TEE during CPB showed no active or residual cysts, though small ventricular septal defects (VSDs) were observed but not addressed due to stable hemodynamics. An intra‐aortic balloon pump (IABP) was placed to mitigate postoperative complications.

**FIGURE 5:**
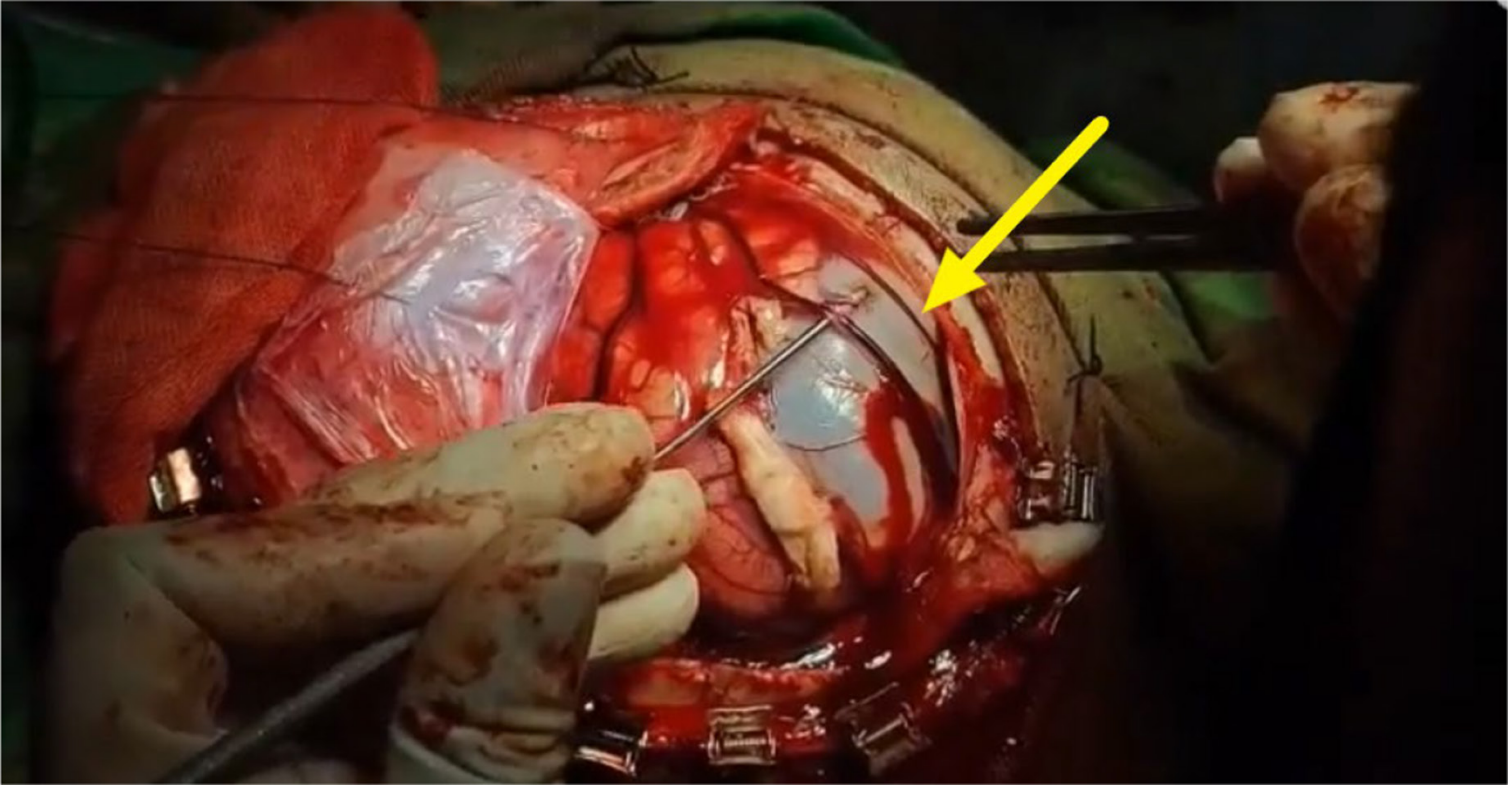
Cerebral craniotomy of a patient with recurrent multi‐organ hydatid cyst. This intraoperative image shows a cerebral hydatid cyst being surgically removed via craniotomy. The yellow arrow indicates a hydatid cyst membrane or capsule, which is being carefully dissected from the surrounding brain tissue. The cyst appears intact, and the surgeon is taking precautions to avoid rupture, which is critical in preventing spillage of cyst contents and potential anaphylaxis.

Approximately 4 weeks later, on November 2, 2019, a cerebral craniotomy was performed to remove recurrent hydatid cysts (Figure 5).

### 2.4 Postoperative Care

The patient was discharged on 17th November 2019, under medical oversight with a regimen including albendazole (400 mg/twice a day) and Levetiracetam (Levebel; 500 mg/once a day; for 6 months), to mitigate the risk of unintentional seizure after brain surgery. Periodic follow‐up appointments were scheduled to monitor the pelvic hydatid cyst, eliminating the need for additional surgical measures.

### 2.5 Present Evaluation

In May 2023, the patient returned solely presenting dyspnea and atypical chest pain, devoid of cerebral manifestations. Cardiac evaluations revealed the recurrence of CHCs without any daughter cysts. Upon physical examination, the patient exhibited normal heart sounds, unremarkable pulmonary auscultation, and a heart rate within normal limits at 70 beats per minute.

### 2.6 New Imaging Insights

**FIGURE 6:**
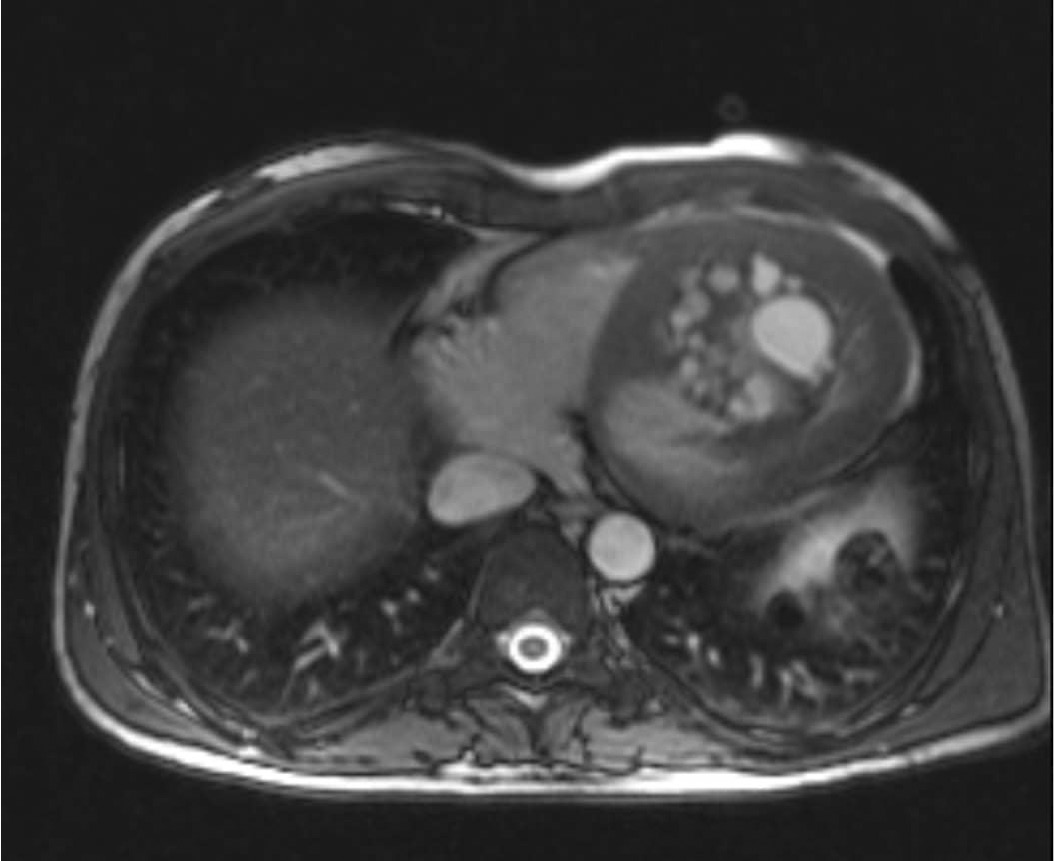
Axial T2‐weighted CMR of a 35‐year‐old male with multi‐organ hydatid cyst. There is an intracavitary multiloculated cystic lesion with a high signal content of cysts and a thin hypo‐signal wall occupying a large part of the lumen of the LV. CMR, cardiac magnetic resonance imaging; LV, left ventricle.

- **Brain CT scan**: revealed No significant findings suggestive of cerebral hydatid cyst recurrence were observed.
- **CMR**: Displayed an enlarged LV size indicating LVH with an LV ejection fraction (LVEF) of 25%–30%. Conversely, the RV size appeared normal without evidence of RVH, with an RVEF of 52% (Figure 6).

- **Echocardiography**: TTE revealed severe left ventricular enlargement with an EF of 30%. Notably, a large multilobulated cystic mass was discerned within the LV, occupying a substantial portion of the LV cavity with an intracavity gradient of 45 mmHg. Mild to moderate mitral regurgitation (MR) was noted, alongside a systolic pulmonary artery pressure (SPAP) of 28 mmHg. Pulmonary embolism was ruled out.
- **Abdominopelvic Ultrasonography:** Abdominopelvic ultrasonographic examination unveiled a significant multi‐cystic lesion within the pelvic cavity, measuring 65 mm × 62 mm, in diameter.

### 2.7 Laboratory Data

ELISA serological tests yielded positive results for *Echinococcus granulosus*, indicating an IgG antibody Titer of 1:640, corroborating the diagnosis of hydatid cyst recurrence.

### 2.8 Treatment

**FIGURE 7:**
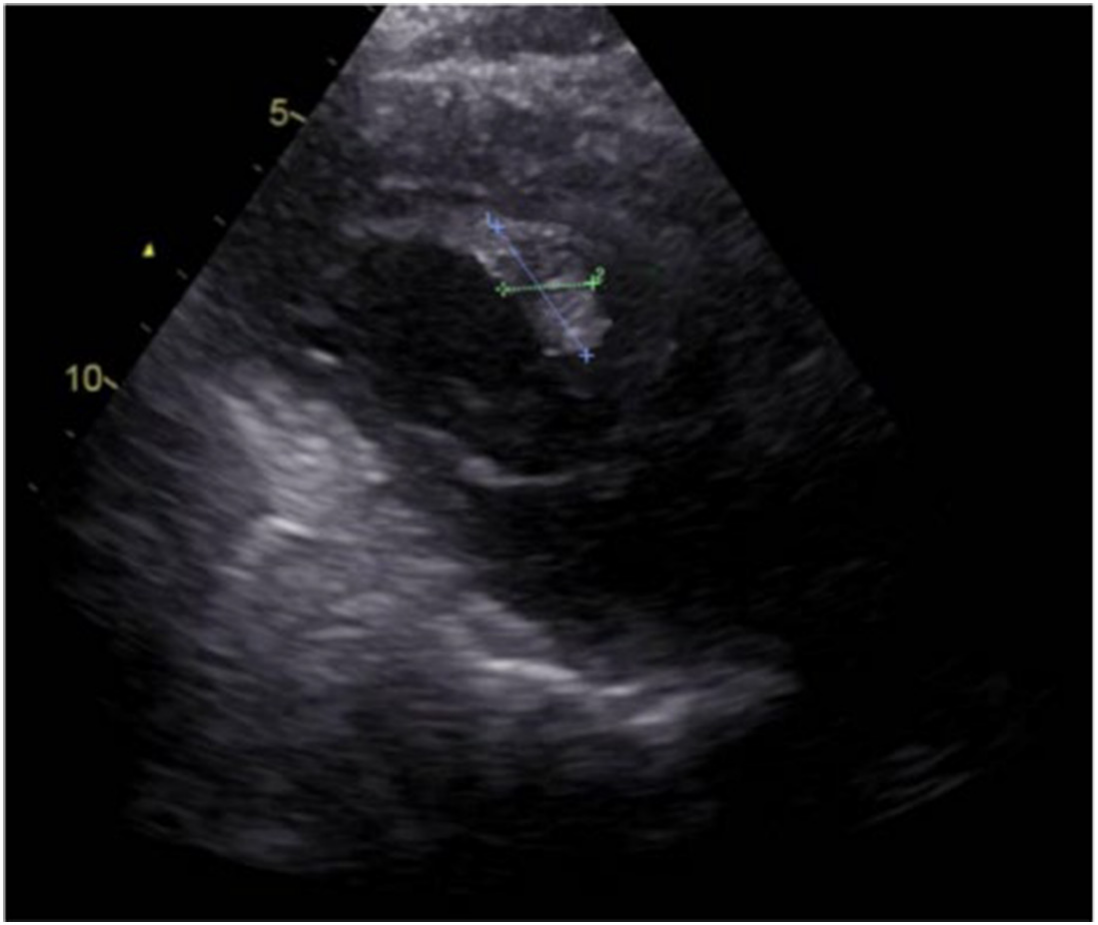
TTE of LV; 6 months after treatment with albendazole and praziquantel in cardiac hydatid cyst. The cystic area decreased in size (2.3 × 2.1 cm), with some evidence of increased echogenicity suggestive of calcification or fibrosis. There are no apparent regional wall motion abnormalities, indicating preserved LV function. LV, left ventricle; TTE, transthoracic echocardiography.

Due to the cyst's inactivity and the recurrence in multiple organs, repeat cardiac surgery was not considered. Given the failure of previous albendazole monotherapy, despite the good adherence and tolerability, the patient was started on combination therapy with albendazole (400 mg twice daily) and praziquantel (40 mg/kg per day) twice weekly for 4 weeks. This regimen was repeated for three courses with a 2‐week interval between each one (until October 2023). Six months after treatment cessation, in April 2024, cardiac symptoms had improved, and echocardiographic imaging revealed significant improvement with one single cyst visualization (2.3 × 2.1 cm) (Figure 7). Abdominopelvic radiological and sonographic assessments indicated reduced size and hyper‐density in cystic lesions, demonstrating partial positive progress. Further follow‐up (to September 2024) via telephone interview with the patient revealed a stable clinical condition, with no signs or symptoms of the initial disease complaints, and no evidence of recurrence. However, recent imaging and other new paraclinical findings are unavailable due to the difference in the treatment center currently overseeing the patient's care.

## 3 Discussion

CHCs are rare but potentially fatal, with varied clinical presentations. These cysts can lead to serious complications like arrhythmias, myocardial infarction, and even unexpected death [11]. Diagnosis is challenging due to atypical symptoms and late detection, often requiring multi‐modality imaging for accurate localization [12]. Patients may present with symptoms like chest pain, palpitations, dyspnea, and syncope [13]. Additionally, the presence of multiorgan involvement, such as hepatic hydatid cysts, underscores the systemic nature of the disease. Early diagnosis and prompt surgical intervention are crucial in managing CHCs to prevent severe complications and improve patient outcomes [12].

Traditionally, three main treatment modalities have been employed: surgical intervention, percutaneous treatment methods such as PAIR (Puncture, Aspiration, Injection, and Reaspiration), and medical therapy utilizing anti‐parasitic drugs [14]. While surgery has historically been the primary approach, its applicability is often constrained by factors such as the patient's clinical status and the location of the hydatid cysts [14].

Cardiac surgery is frequently complicated by postoperative arrhythmias, such as AF, which can lead to increased morbidity and mortality [15]. The study by Marah Jamli et al. [16] underscores the significance of recognizing and managing postoperative rhythm disturbances, particularly AF, which may arise, especially in cases with cardiac hydatid septal involvement. The utilization of IABPs in the prime treatment of our case illustrates a strategic approach to enhancing myocardial perfusion and cardiac output, indirectly mitigating the risk of potential conduction disorders postoperatively.

Percutaneous methods like PAIR have gained recognition; however, their utility may be restricted by contraindications and complications, especially in cases involving cysts located in vital organs like the heart or brain [17].

In recent years, medical therapy has emerged as a viable alternative, particularly in cases where invasive procedures pose risks or are not feasible. Preoperative medical treatment aims to reduce the size of hydatid cysts, sterilize them, and minimize the risk of relapse. Moreover, for disseminated hydatidosis, medical therapy constitutes the sole therapeutic option [18, 19, 20].

The integration of adjunctive medical therapy with benzimidazole derivatives, such as albendazole or mebendazole, has become increasingly prominent both pre‐ and postoperatively to mitigate recurrence risk. These medications exhibit efficacy in inhibiting cyst growth and preventing postoperative recurrence, thereby complementing surgical interventions [9]. While other anthelmintic chemotherapies such as praziquantel and nitazoxanide have been investigated, their effectiveness against *Echinococcus* spp. remains inferior to that of benzimidazoles [9]. Nevertheless, observational studies suggest that combining albendazole with praziquantel may enhance cure rates compared to albendazole alone, highlighting the potential synergistic activity of combined therapy [9].

A safety analysis in 57 cases with hydatidosis treated with albendazole plus praziquantel revealed generally favorable outcomes [21]. The safety analysis included self‐reporting of adverse events, hemograms, and biochemistry before and after treatment. Only eight (14%) patients reported some mild adverse effects, with the most frequent being digestive symptoms. These adverse events tended to occur within the first 2 weeks after the start of treatment, with no clinically relevant changes in hematological results detected during the treatment period. These findings highlight the overall tolerability of combined therapy and support its use in the management of hydatidosis.

In a cross‐sectional quasi‐experimental study with nonrandomized sampling, by Mahin Jamshidi et al. [9], nine cases with multi‐hydatid cysts were treated with albendazole and praziquantel and followed up for an average of 18 months. Clinical and radiological responses were evaluated for partial to complete response. Results revealed Symptoms disappeared in seven (77.8%) patients and improved partially in two (22.2%) patients, and Radiological assessment showed significant improvement in five (55.6%) and partial improvement in four (44.4%) patients.

Moreover, a 3‐month combinational therapy (albendazole and praziquantel) on 10 patients diagnosed with hepatic hydatid cyst revealed complete radiological remission and no recurrence of symptoms in 1‐year follow‐up [22].

Furthermore, Yasawy et al. [23] demonstrated that the concurrent administration of albendazole and praziquantel for the treatment or preoperative chemoprophylaxis of hydatid cysts resulted in a reduction in treatment duration compared to using each medication individually.

Similar to our case, a 4.5‐month co‐administration of albendazole (400 mg/twice a day) and praziquantel (40 mg/kg per day), accompanied by a 6‐month follow‐up demonstrated a significant decrease in size and morphology of CHC and a considerable partial positive progress in abdominopelvic lesions. Also, no adverse reactions were reported through the treatment and follow‐up period (up to 11 months), which reinforces the prospective use of this combination therapy in the treatment of extensive hydatid cysts (cardiac and multi‐organ). Table 1 summarizes key studies comparing the efficacy of albendazole monotherapy versus combination therapy with albendazole and praziquantel in the treatment of hydatid cysts.

**TABLE 1:** Comparison of albendazole monotherapy and albendazole plus praziquantel combination therapy for hydatid cysts.

Study	Patient/animal model	Treatment regimen	Results for albendazole alone	Results for albendazole + praziquantel	Conclusion
Mohamed et al. [24]	41 human patients with hydatid disease	Albendazole: 22 patients, combination: 19 patients	36.4% complete cyst disappearance, treatment duration: 6 months to 2 years	47.4% complete cyst disappearance, treatment duration: 2–6 months	Combination therapy more effective with a shorter treatment duration
Jamshidi et al. [9]	Nine human patients with multiple hydatid cysts	Albendazole: 400 mg twice daily, praziquantel: 40 mg/kg twice a week for 4 weeks (three courses with 2‐week intervals)	Not applicable (only combination therapy used)	55.6% significant improvement in radiological assessment, 77.8% symptom disappearance	Combination therapy is effective as an alternative to surgery
Yasawy et al. [22]	Four human patients with hydatid disease	Albendazole: 400 mg twice daily, praziquantel: 50 mg/kg in different regimens	Not applicable (only combination therapy used)	75%–100% cyst disappearance within 3 months	Combination therapy provides rapid and effective cyst reduction
Popova et al. [25]	20 human patients with hepatic and/or pulmonary hydatidosis	Albendazole: 15 mg/kg/day, praziquantel: 40 mg/kg weekly for 2–9 months	Not reported separately	85% improvement or cure observed via imaging	Combination therapy appears effective, with no significant side effects
Rafiei et al. [26]	30 mice with experimental hydatidosis	Albendazole: 50 mg/kg/day for 4 weeks	63.78% reduction in cyst number, 79.39% reduction in cyst weight	91.70% reduction in cyst number, 90% reduction in cyst weight	Combination therapy is significantly more effective in reducing cyst development
Haralabidis et al. [27]	70 human patients treated after surgery	Albendazole: various doses	Statistically significant reduction in cyst shrinkage and calcification	Enhanced shrinkage (over 80%) and calcification with combination therapy	Combination therapy improves long‐term outcomes, especially post‐surgery

### 3.1 Clinical Challenges and Limitations

This case highlights significant clinical challenges in managing multiorgan hydatid disease, particularly in the context of recurrent cardiac involvement. A major limitation is the unavailability of recent imaging and paraclinical data from the patient's current treatment center, which restricts comprehensive evaluation of the long‐term efficacy of combination therapy with albendazole and praziquantel. Additionally, the reliance on telephonic follow‐up introduces potential bias, as the absence of clinical symptoms may not accurately reflect disease recurrence or progression, especially in the context of complex, multiorgan hydatid cysts. While the decision to avoid further surgical intervention was driven by cyst inactivity and high surgical risk, this underscores the broader challenge of balancing conservative management with the need for definitive treatment, particularly when surgery remains the gold standard for hydatid cysts. These factors highlight the necessity for larger studies to refine treatment protocols and better assess long‐term outcomes for similar cases, especially comparing albendazole alone and in combination with praziquantel.

## 4 Conclusion

Our case emphasizes the potential of combination therapy with albendazole and praziquantel as a viable alternative to surgical intervention in complex and high‐risk multiorgan hydatid disease. Early multimodal imaging and careful clinical monitoring are crucial in optimizing treatment outcomes and minimizing the need for invasive procedures.

## Author Contributions

**Soroush Najdaghi:** investigation, writing – review and editing. **Azin Alizadehasl:** project administration, supervision. **Narguess Abbaszade:** investigation. **Seyedeh Fatemeh Hosseini Jebelli:** writing – original draft. **Delaram Narimani Davani:** writing – original draft. **Azam Yalameh Aliabadi:** methodology, resources. **Mehrdad Haghazali:** resources, visualization. **Alireza Yaghoubi Gloverdi:** resources, visualization.

## Ethics Statement

Written informed consent was obtained from the patient for the initiation of the therapy.

## Conflicts of Interest

The authors declare no conflicts of interest.

## Data Availability

The data that support the findings of this study are available from the corresponding author upon reasonable request.
